# Supplementary material for: Are carbon emissions trading and green financial instruments synergistic? -Comprehensive quantitative research based on content analysis
Source: PLoS One. 2024 Mar 7;19(3):e0298601. doi: 10.1371/journal.pone.0298601 (PMC10919601; doi:10.1371/journal.pone.0298601)
Supplement: S1 Data — (ZIP) [file pone.0298601.s001.zip › Data/地级市Z_F工作报告.zip!/┴╔─■/│»╤⌠╩╨/└┤╫╘╣1⁄2╓┌║┼í╛┬φ┐╦╩2╛▌═°í┐_2015.docx]

各位代表：

现在，我代表市人民政府向大会报告工作，请予审议，并请各位政协委员和其他列席人员提出意见。

一、过去一年工作回顾

2014年，是朝阳发展极不平凡的一年。一年来，面对经济下滑、旱灾爆发、多重矛盾交织等诸多严峻考验，我们在省委、省政府和市委的正确领导下，在市人大及其常委会和市政协的监督支持下，主动适应经济新常态，抢抓新一轮东北振兴等重大历史机遇，取得了改革开放、经济社会发展的新成绩。

——经济总体保持平稳，并实现稳中有增、稳中趋优，预计完成地区生产总值1020亿元，增长4.5%。

——抓改革、促调整取得积极成效，政府简政放权、五大园区改革全面铺开，经济结构出现积极变化，服务业增速首次较大幅度超过经济增速。

——一批重点产业、民生保障、生态环境、社会事业项目扎实推进，预计完成固定资产投资836.7亿元，增长2%。

——我市成功晋升省级环保模范城市。市区空气质量优良天数达标率超过80%，排名进入全省前五名。

——居民收入保持较快增长，预计城镇和农村常住居民人均可支配收入分别实现19815元和9966元，分别增长10%和13%。就业、社保、扶贫、集中供热等民生工作取得新成果。

各位代表，过去一年，面对前所未有的困难和持续加大的经济下行压力，我们深入学习贯彻习近平总书记系列重要讲话精神，坚持稳中求进、改革创新，主要做了以下几方面工作：

一是全力抓好稳增长。为解决工业主导产业受制、房地产供需“两端疲软”问题，我们坚持问题导向，高密度开展现场办公，解决实际问题，先后出台21条促进经济增长、9条保持房地产业发展的新政策，调控经济运行，增强市场信心，稳定社会预期。特别在企业发展遇到困难的情况下，通过落实项目包扶责任制、支持重点企业搞好搞活生产经营、采取结构性减税和普遍性降费等办法，支持实体经济发展。全年共减轻企业负担7.47亿元。凌钢开拓国际市场取得重大进展，出口创汇实现翻两番；鞍凌钢重组注资后，资产负债率大幅下降，发展出现新转机。

受各种因素影响，财政收入出现了多年少有的负增长。针对这种情况，我们积极创新财税征管工作，调整优化税收结构，加强对骨干税源服务及零散税源征管，不断做实财政收入。市本级实现公共财政预算收入14.1亿元，增长7.8%。全市完成公共财政预算收入101.7亿元，下降9.6%。

二是着力深化改革开放。在经济运行遇到困难的情况下，我们依靠改革的办法和市场的力量，积极推进22项重点领域改革，重塑发展动力。

通过简政放权、清理审批前置要件，房地产、工业、政府投资类项目审批用章压缩2／3，审批时限控制到一个月以内。主动承接国家和省下放审批157项，向县区放权145项，食药监、工商质监管理体制调整和卫生计生系统整合全面完成。大力解决中介机构垄断经营问题，15户企业与挂靠的机关事业单位实现脱钩。工商登记制度改革后，新注册登记企业2500多户，增长85%，形成了新的创业热潮。强力实施低保核查、公益岗清理整顿，加强二手车交易和成品油市场监管，一批群众反映强烈的突出问题得到有效解决。市区财政体制改革成效显现，市直单位财务统管改革全面完成。这些不仅为市场松了绑、为企业发展助了力，也为全面深化改革赢得了体制优势和保障。

用全新的体制机制，整合组建了燕都新区、高新区和凤凰山、鸟化石、牛河梁景区，赋予市级经济管理权限和必要的财政、人事管理权限，五大园区改革迸发出新活力，开始走上市场化运作、封闭式管理的发展轨道。

加快推进招商引资体制机制创新，组织开展了赴台湾、香港、上海、北京等12次大型招商活动，引进域外资金295亿元，增长21.3%；实际利用外资2.5亿美元，增长20%；外贸出口6.3亿美元，增长26%。

三是坚定不移调结构。针对产业发展中长期存在的结构性问题，我们利用市场倒逼机制，补短板，促提升，向转型要发展，向调整要出路。

积极打造工业五大主导产业。在稳定调优冶金业的同时，加快推进汽车及零部件、农产品及食品加工、能源及新能源、建材及设备制造等产业培育，重点项目和产业集群建设取得新进展。上海飞和实现整车下线，大连阳光世纪卡玛斯重卡项目签约落地，朝阳人民期盼的“汽车梦”变成现实。盛泽页岩油、中智力拓镀膜玻璃、新浙锰业高碳锰铁、旗盛粉末冶金、紫陶建材等调结构项目实现投产，立塬新能源、天翼国基高纯合金、金麟高纯铁粉等高科技项目加速成长。

大力发展现代农业。克服历史罕见旱灾影响，因势利导“以旱促调”，粮食产量实现近40亿斤。深入实施设施农业提质增效工程，通过推广新技术、加大农药残留检测、搞好市场流通等措施，设施农业产值实现158亿元。新增节水滴灌面积51.8万亩、畜禽标准化养殖小区215个，完成经济林52万亩、草原沙化治理57万亩。各地农产品及食品加工园区建设实现良好开局。农村土地确权登记颁证试点全面完成。人工影响天气飞机作业保障分中心启动建设。

实施服务业四年行动计划。积极培育旅游地接市场，神龙漂流度假村、龙凤山景区、天富雪上乐园等项目建成开业，旅游影响力进一步提升。城区“8+2”商贸市场开工建设，各类农产品流通中心、交易基地和特色市场建设加快推进。市电子商务产业园区投入运营，30户电商企业实现入驻。新增贷款和直接融资均突破110亿元，16户企业成功在上海股权托管交易中心挂牌，朝阳银行沈阳分行、本溪分行挂牌营业。

四是加快推进城乡建设。我们坚持遵循发展的规律性、避免盲目性，注重抓好新城新区基础设施建设，积极利用棚改和开行贷款政策，加快推进城中村、城郊村和重点镇改造，城镇化率达到45.2%，提高2个百分点。

京沈客专朝阳段征地拆迁工作高质量完成。国电热电联产、燕阳山大桥开工建设。全长75.2公里的燕山湖至白石水库滨河路全线通车，城区路面最宽、配套设施较完善的新主干道——文明路建成投入使用。宜居乡村建设、农村环境综合整治、危房改造全面展开，农村面貌明显改观。

中心城区控制性详规编制启动实施。新建和改造市县两级城市道路41公里、排水管网106公里。城市南部排水干渠基本竣工，大凌河城区段两岸亮化工程初步建成。市容环境集中整治深入开展，城市管理更加规范有序。

持续推进青山、碧水、蓝天工程，城区环城绿化带建设、凤凰山采石场植被恢复工程启动实施，燕阳山公园、高速路出口及周边迎面山绿化扎实推进。完成植树造林105.3万亩，水土保持生态治理91.4万亩。大小凌河流域“三带”规划高标准完成，流域治理深入开展，退田还河、重点防洪和生态景观工程建设走在全省前列，新建成的大凌河音乐喷泉、凌河湾湿地成为城市靓丽美景。4座空气监测站实行日监测发布，7座污水处理厂和19家国控重点污染源企业实现在线监测。

五是保持民生和社会和谐稳定。我们把有限财政资源用在刀刃上，紧兜民生底线，推动社会事业发展，提高社会治理水平。

投入资金12.7亿元，完成了十大重点民生工程、37件具体民生实事。其中，实现实名制就业5万人，登记失业率控制在3%以内。建立统一的城乡居民养老保险制度，企业退休人员养老金、城镇居民基本医疗保险待遇、城乡低保、新农合人均筹资标准进一步提高，各类保险覆盖面不断扩大。扶贫示范村建设、移民搬迁工作扎实推进。完成老旧小区改造240万平方米，13.5万农村人口饮水安全问题得到解决，农村亮化工程实现全覆盖。特别是克服筹资难、工期短、热源整合棘手等重重困难，城区南部16万居民用户、1400万平方米实现了集中供暖，供暖质量稳步提高，拆除停用锅炉房67座，减排二氧化硫2048吨、烟粉尘1.7万吨。

十分钟便民服务圈建设全面铺开，城市社区老人日间照料室全部建成，老旧弃管小区物业管理不断规范，“四大九小”服务圈逐步形成。

新建改扩建20所标准化幼儿园，完成200所义务教育学校标准化建设，普通高中优质特色办学取得新进展，城区中职教育专业资源完成优化整合。120急救指挥、药品采购、基层卫生信息化“三大平台”建成投入运行，县级公立医院改革加快推进。科技创新服务平台建设初见成效。文艺创作成就显著，成功举办群众文化节、全民健身节和国民体质监测等活动，我市在省十二运首次进入金牌榜前三名。国土资源管理水平和保障能力显著提高。人民调解、社区矫正、法律援助工作取得新成效，综治维稳、严打整治、道路交通、市区禁放烟花爆竹管理形成常态化。信访形势持续好转，摘掉了全省“信访大市”帽子。持续开展隐患排查和整改，安全生产形势总体平稳。加强消防设施投入和应急救援能力建设，火灾防控水平不断提高。国防动员、人防和民兵预备役建设进一步加强，双拥创建工作扎实推进。民族团结进步事业不断发展。住房公积金、档案、应急管理、国家安全、外事侨务、援疆援青以及老年人、妇女儿童、残疾人权益保障等工作也取得了新成绩。

过去一年，我们深入开展党的群众路线教育实践活动，政风建设取得积极成效。坚持依法行政，自觉接受人大及其常委会的法律监督和工作监督，主动接受政协的民主监督，共办理人大代表建议62件、政协提案213件，满意率均达到98%以上。畅通群众诉求渠道，扎实推进政务公开和市民诉求办理工作，群众对政府工作的满意率明显提升。坚决贯彻执行中央“八项规定”，规范权力运行，政府投资项目资金审减额达1.3亿元，市本级“三公”经费支出按可比口径下降24.3%。

七个县（市）区一年来也取得了可喜的发展成果：

北票市粉末冶金、除尘装备、能源建材等产业集群不断壮大，台吉新区和老城改造加快推进，1.1万户棚改居民喜迁新居，百丽购物广场、伊斯曼温泉小镇投入运营，村庄环境治理卓有成效，并荣获省级文明城市和日光温室番茄一县一业示范县称号。

凌源市进入国家现代农业示范区和全国产粮大县行列，并获得日光温室黄瓜生产全国第一县称号。汽车、玻璃两大产业发展掀开新篇章，利浩型材二期等项目快速推进。永利广场开盘销售，兴隆大家庭开槽动工。评剧《凌水湾》获国家和省优秀剧目奖。

朝阳县三次产业结构调整加快推进，东鑫精炼镍、东锆核级锆、奥瑞德宝马汽车配件、皓盛集团综合体等项目陆续进入投产期，工业园区加速向现代物流园区转型，新县城整体搬迁全面启动，并荣获全省小凌河流域农产品质量示范基地称号。

建平县成功入围全国首批农村职业教育和成人教育示范县。大旱之年粮食产量达到16亿斤，全国产粮大县地位得到巩固。粉末冶金生产基地、非金属精细加工园区启动建设，纳鑫粉末冶金、华潍膨润土等资源精深加工项目开工建设。

喀左县再获国家卫生县和全国民族团结进步模范县称号。产业结构调整、园区建设、凌河治理等工作走在全市前列。冶金铸锻集群加快转型升级，紫陶建材集群投资实现倍增。财富领域购物广场、紫砂古玩化石文化市场建成投入运营。

双塔区金属新材料产业集群核心项目建设扎实推进，凤凰文化旅游集聚区晋升为省级重点现代服务业集聚区。哨口城中村改造全面启动。电子商务产业园投入运营。以棚果采摘、民俗文化、湿地旅游为主的都市农业休闲旅游带初具雏形。

龙城区坚持新方法、严标准推进征收拆迁，几项重点拆迁任务高质量完成。佛瑞达三期和朝重、重机、黑猫炭黑搬迁改造加快推进，新天地商贸文化城正式营业，生态农业温泉度假村、庙子沟滑雪场续建项目投入运营，禽蛋出口被评为省级示范区。

各位代表，一年来的实践告诉我们，朝阳人民勤劳智慧、自强不息、渴望发展，只要大家心往一处想，劲往一处使，再大的困难也阻挡不住我们前进的脚步。一年来的实践提醒我们，解决长期积累的体制机制矛盾不会一蹴而就，我们必须有充分的心理准备，必须保持干事创业的激情和长期艰苦奋斗的韧性。一年来的实践昭示我们，在发展的道路上，不会是一片坦途，我们必须抢抓新机遇，主动适应、科学引领经济新常态，把朝阳发展一步一个脚印地推向前进。

过去一年取得的成绩，是省委、省政府和市委坚强领导、正确决策的结果，是市人大、市政协以及社会各界大力支持、鼎力相助的结果，是全市人民团结一心、共同努力的结果。在此，我代表市政府，向各个岗位上辛勤耕耘的全市各族人民，向给予我们监督支持的人大代表、政协委员、各民主党派、工商联、无党派及社会各界人士，向情系朝阳、支持朝阳的驻朝中省直单位、解放军指战员和武警官兵，表示诚挚的感谢！

同时，我们也清醒认识到，经济总量小、民生水平低、产业结构不优、科技创新能力不足等老问题尚未解决，新问题又在显现：一是经济增速出现回落，低于全国和全省平均水平，部分指标下降幅度较大；二是结构调整阵痛显现，铁选冶金业陷入低谷，企业生产经营困难加剧；三是房地产、政府债务、财政收支等潜在风险增大；四是“四风”整治虽取得初步成效，但基础尚不稳固，政府软环境、干部作风和廉政建设仍需进一步加强。对这些问题，我们将采取切实有力措施，认真加以解决。

二、2015年工作安排

各位代表，今年是全面深化改革的关键之年，是推进依法治市的重要之年，是完成“十二五”规划的收官之年，做好政府各项工作意义重大。当前，朝阳发展与全国、全省一样，正处在三期叠加效应的特殊时期、各种矛盾相互交织的复杂时期、经济持续下行的严峻时期，形势不容乐观，我们必须防微虑远，保持定力，牢牢把握发展的主动权。同时也要看到，随着新一轮东北振兴、京津冀协同发展、“一带一路”开放等国家战略和新一轮突破辽西北战略的实施，多重发展机遇已形成相互叠加、融合带动之势。特别是最近，党中央作出了经济发展新常态的重大战略判断，我们一定要结合实际，准确把握新常态的特征和规律，抢抓各种机遇，打造新的发展引擎，寻求新的增长空间，努力走出一条符合朝阳实际的科学发展、可持续发展和包容性发展之路。

今年政府工作的总体要求是：全面贯彻落实党的十八大和十八届三中、四中全会精神，按照市委九届八次全会暨经济工作会议要求，坚持稳中求进工作总基调，坚持以提高经济发展质量和效益为中心，主动适应经济发展新常态，积极抢抓各种政策机遇，围绕实施“五大战略”、建设“五大基地”目标，大力推进经济、政治、文化、社会、生态文明建设，努力实现经济持续健康发展和社会和谐稳定。

今年的主要预期目标是：地区生产总值增长6.5%左右，固定资产投资增长7%左右，公共财政预算收入增长2%左右，社会消费品零售总额增长12%左右，城镇和农村常住居民人均可支配收入分别增长10%和11%左右。

（一）保持经济平稳健康运行

把稳投资、稳增长作为首要任务，稳住经济运行，保证经济增长处于合理区间。

开展项目建设年活动。认真对接落实国务院28号文件，推进与央企深度合作，激发民间投资活力，确保实施亿元以上项目350个，完成投资600亿元以上。建立更有效的项目考核机制，重点加大当年开工项目考核力度，确保新形成的投资是有效投资、精准投资，真正投到符合朝阳长远发展的行业和产业上。

抓好经济运行组织协调。主动适应新常态下经济运行的新趋势、新变化，坚持问题导向，深入落实重点企业和项目包扶责任制，加强经济运行分析监测，抓好各种生产要素协调保障，增强经济运行的稳定性。

全力支持企业发展。研究制定推动重点行业、重点企业恢复性增长的政策，积极争取中电投燕山湖电厂二期项目，加快推进鞍钢朝阳钢铁公司新上100万吨钢填平补齐项目，支持凌钢调整产品结构、降低资产负债率、扩大上市融资。着力解决民营企业在创办、成长、创新、税费、融资等方面存在的实际问题，帮助经营困难的中小微企业开拓市场、降低成本、走出困境，确保新增销售收入超10亿元企业2户、超亿元企业15户，规模以上工业增加值增长5%左右。

多措并举促进消费。通过增加收入、完善政策、改善环境，加大对新产业、新业态、新模式支持力度，培育新的消费热点，确保服务业增速高于经济增速。

（二）加快推进各项改革

开展园区（开发区）改革建设年活动。创新管理体制机制，积极搭建融资和管理服务平台，把五大园区打造成改革创新的先导区、产业转型的示范区、经济增长的迸发区。燕都新区要树立产业兴区理念，扎实推进金属新材料、高端装备制造、高科技电子等产业集群发展，加快实现产城融合。高新区要围绕重点企业和产品，抓好定向招商和以商招商，尽快在新能源汽车项目引进上实现突破。凤凰山、鸟化石、牛河梁三大景区要充分发挥在文化旅游牵动战略中的示范带动作用，以市场为导向深化改革，突出抓好核心项目建设和环境打造，积极引进知名企业和战略投资者，尽快闯出旅游业发展新路。各县（市）区要全力建设体制机制创新的工业园区、农产品及食品加工园区和文化旅游景区，避免产业同质化趋向，争取2年内实现省级园区全覆盖。

深入推进政府简政放权、放管结合改革。积极抓好市县两级政府机构改革和职能转变。大幅减少前置审批，确保前置审批要件精简50%以上。把精简审批公章、压缩审批时限全面落实到位。抓好审批权限的承接和下放，积极推进投资项目网上核准。探索推行政府和社会资本合作（PPP）等多种投融资模式，与简政放权形成组合拳。加强中介机构规范管理，加大社会组织整顿力度，提升中介行业整体水平。落实普遍性降费要求，实施涉企收费目录管理，规范殡葬、教育、医疗等民生收费。搞好市场监管和信用体系建设，严格落实企业不良行为记录“一次黄牌警告、两次红牌退出”制度，打造信用朝阳。建立权力清单、负面清单和责任清单制度。深化预算管理制度改革，完善政府预算体系，改进预算管理方式，推进预决算公开。全面启动“三证合一”改革。

抓好招商引资体制机制创新。积极调整招商方式和重点，面向京津冀、上海等地区和俄、日、韩等周边国家，面向全国500强和行业龙头企业，形成专业化、系统化招商体系。扶持和推动外贸企业发展，不断扩大出口规模。域外引资和出口创汇分别增长15%和20%左右。

（三）抓好产业结构调整升级

利用市场倒逼机制，推动传统产业向中高端迈进，培育发展战略性新兴产业和高新技术产业，增强服务业带动作用。

加快打造工业五大主导产业。积极调优冶金业，抓好铁选企业兼并重组、提档升级，支持天翼国基高纯合金、金麟高纯铁粉、新都黄金搬迁改造、万阳富锰渣、宏跃冷轧带钢、昊天不锈钢、求业钒钛耐磨新材料、旗盛和纳鑫粉末冶金、金工钒钛等高品质冶金项目建设。围绕“凌源整车、高新区新能源汽车”两大龙头，大力发展汽车及零部件产业，支持上海飞和、大连阳光世纪、鹏达铸造、奥瑞德宝马汽车配件、华兴万达和顺达丁基内胎等企业和项目加快发展，打造中国北方“汽车城”。大力发展农产品及食品加工业，在注重培育现有企业的同时，面向涉农央企和国内品牌农事企业进行定向招商、敲门招商。做强能源及新能源产业，积极推进传统能源和风能、太阳能、光伏发电、油页岩加工等新能源产业发展。做大建材及设备制造产业，加快装备制造业转型，支持朝重和重机搬迁改造、波迪环保除尘设备、中智力拓镀膜玻璃深加工、万华轻型墙体、华潍膨润土、紫陶建材等项目建设。要发挥好这些核心项目的带动作用，接长产业链条，吸引配套企业集聚，提高产业集中度和丰厚度，争取通过3—5年的调整培育，逐步形成工业多点支撑的新格局。

加快实施服务业四年行动计划。发挥现代服务业在调结构中的带动作用，推动生产性服务业与制造业、生活性服务业与社会事业融合发展。把旅游业作为新常态下一项战略性增长点和调结构、促升级的重要抓手，科学编制旅游业发展规划，加强旅游基础设施建设，加大宣传推介力度，搞好地接市场培育和客源地市场拓展，加快实现旅游业与其他产业深度融合，形成扩大就业、改善民生、拉动消费的新引擎。坚持消化存量、调优结构，引导房地产企业由普通住宅向商业、工业、旅游、养老等地产业态转型。完善商品市场和现代物流体系，加快推进东北亚农商物流城、龙城众鑫冷链物流、朝阳县柳城物流园、建平惠通农产品物流中心、北票庄头农产品加工流通园区、喀左紫陶物流园等项目建设。加强电子商务产业园规范管理，搞好北方矿产品、朝阳农产品交易平台建设，推动传统产业利用电商转型，争创省级电子商务集聚示范园区和东北地区电子商务重点市。搞好金融市场体系建设，新增贷款110亿元，实现3—5户企业在新三板挂牌和村镇银行县域全覆盖。大力发展通用航空、文化产业、健康养老等新型服务业。

（四）加快转变农业发展方式

抓好“三农”工作，大力发展现代农业，让农业经营有效益，成为有奔头的产业。

深入实施设施农业扩量提质增效工程，加强技术服务、市场流通、质量安全等工作，积极申报国家现代农业示范区和现代设施农业示范区；新增节水滴灌面积71.4万亩，总量达到200万亩；完成25万亩经济林建设，大力发展畜牧业标准化养殖，推动农业走上产出高效、产品安全、资源节约的现代农业发展道路。

大力发展农业产业化，加快各地农产品及食品加工园区建设，抓好龙头企业扶持和引进，加大涉农资金整合力度，支持龙头企业发展，引导鼓励现有企业通过与国内大企业合资合作、股权转让等方式不断发展壮大，努力把产业链、价值链等现代产业组织方式引入农业，促进农业与其他产业融合互动。发挥农村供销社作用，进一步健全完善农村市场流通体系。

深化农村改革，全面推进农村土地确权登记颁证工作，促进土地经营权有序流转。积极发展多种形式适度规模经营，支持种养大户、家庭农场和农民合作社等新型农业经营主体发展。

（五）加快推进新型城镇化建设

在注重城镇化率的同时，把工作重心转到提高城镇化质量和内涵上，加快户籍制度改革和基本公共服务均等化。抓好各新城新区基础设施和配套功能建设，促其走上良性发展轨道。推进棚户区和城中村、城郊村、重点镇改造，新实施棚户区改造1.5万户，完成城中村改造350万平方米、小城镇建设200万平方米。搞好一县一个新市镇试点建设，争取每个县（市）都建成3—5个特色小城镇。

提升建设管理水平，抓好城市地下管网、老旧社区设施改造。打通友谊大街南段，新建文昌路，建设文化路、黄河路两座公铁立交桥，缓解城市交通拥堵状况。完善城区南部集中供热改造项目，启动建设北部集中供热主管网。继续开展市容环境集中整治行动。

启动实施京沈客专两条连接线、锦白货运专线和锦承线朝阳段、叶赤线扩能改造征地拆迁工作，推进朝巴线、京沈线等国省干线改造，抓好县乡公路养护规范化管理。配合省里搞好辽西北供水及配套工程建设，完成燕阳山大桥、500千伏利州输变电建设。

（六）建设生态文明的美好家园

继续抓好城区环城绿化带建设，完成5万亩造林绿化任务。加快实施凤凰山采石场植被恢复工程，让被破坏的山体重现绿色。新增植树造林75万亩，全民义务植树900万株，完成水土保持生态治理面积70万亩。

加大凌河流域治理管护力度，推进干流薄弱环节和重点支流建设，抓好一批“三带”精品项目和滨河路两侧绿化景观工程，实现重点流域向全域治理转变，打造凌河治理升级版。

加快“一县一热源”建设，抓好建筑工地扬尘、中心城区周边煤堆料场、城中村小烟囱治理，积极推进钢铁、水泥、电力行业脱硝脱硫等污染减排工程，让群众不断看到治理雾霾新成效。积极推进污水处理设施稳定运行、水质达标和中水回用工作。

加快宜居乡村建设，抓好农村环境综合整治，形成垃圾、污水、畜禽粪便处理常态化，努力建设美丽乡村。

推广智慧公共服务、交通、社区、园区等系统建设，加快把我市打造成“智能化、信息化、网络化”的智慧城市。

（七）抓好十大重点民生工程

一是就业维权工程。新增实名制就业5万人，城镇登记失业率控制在3.5%以内。打击侵害农民工权益的违法行为，拖欠工资清偿率达到90%以上。对登记失业的困难家庭大学毕业生实行兜底就业安置。

二是社会保障工程。新增“五险”扩面3万人次。继续提高企业退休人员基本养老金标准。推进医疗保险市级统筹，实现全市异地就医即时结算。搞好金保工程信息化建设，加快实现社会保险一卡通用。

三是教育助学工程。启动“一乡镇一所标准化幼儿园”五年建设工程，新建改扩建标准化幼儿园15所。抓好薄弱学校改造，完成168所“改薄”、188所校舍维修改造项目建设。异地新建市特殊教育中心，为聋哑儿童提供更好的学习生活环境。新建榆树林、长江路小学，进一步缓解城区学校大班额问题。

四是医疗卫生工程。加快市妇儿医院建设，实现孕优工程全覆盖。推行新农合补偿全部由居民健康卡结算，方便群众就医。

五是扶困救助工程。继续提高城乡居民最低生活保障标准。为全市符合条件的重度贫困残疾人免费适配辅助器具。建立规范的临时救助制度，为特殊困难群体或遭遇特殊变故家庭提供一次性救助。解决8.4万农村人口饮水安全问题。在市本级新建一所综合性公办养老机构，抓好6所养老托管中心运营和一批民办养老机构建设，新增养老托管床位5000张。

六是居住环境改善工程。实施老旧小区改造120万平方米，“暖房子”工程65万平方米。建立乡村专业保洁队伍，加快实现农村垃圾村级“日产日清”。

七是扶贫开发工程。启动第二轮37个综合扶贫示范村建设，完成400户移民搬迁任务，确保10万农村低收入贫困人口实现精准稳定脱贫。

八是十分钟便民服务圈工程。已建成的社区老人日间照料室实现全面运营，城区65%以上老旧弃管小区有物业管理，80%以上社区有便捷的家政服务。加强社区市场规范整顿，实现社区警务和社区调解全覆盖。大力开展群众性科教文体活动，创建文明和谐新社区。

九是文体惠民工程。开展送戏下乡400场，实现社区书屋全覆盖，打造“书香朝阳”。举办文化旅游节、文化艺术节、全民健身节等活动。新建260个全民健身广场和12个公共篮球场。

十是社会安全工程。搞好矿山应急救援基地、消防队站、食品药品检验检测中心建设，对校园周边“小饭桌”实施规范化管理，保障人民群众生命财产和饮食用药安全。放开机动车安检机构市场，破解“检车难”问题。

（八）推进社会事业发展和社会治理创新

搞好义务教育学校标准化建设，创建全国义务教育发展基本均衡县。完善现代职业教育体系，发展现代职业教育。推动朝阳师专办学转型升级。加快县级公立医院改革步伐，全面提高“三大平台”建成后的运行质量。积极推进科技创新服务体系建设。发展公益性文化事业，繁荣文艺精品创作，积极打造博物馆城，加快牛河梁遗址“申遗”进程，启动辽西文化生态保护区创建工作，争创国家历史文化名城和省文化旅游改革试验市。促进群众体育、竞技体育和体育产业全面发展。高质量完成“十三五”规划编制工作。抓好统计、档案、地方志、地震等各项工作，发展慈善、老龄、妇女儿童、关心下一代等事业。

深入开展法制宣传教育，自觉运用法治思维和法治方式深化改革、推动发展。加强安全生产和人员密集场所安全管理工作，抓好矿山和尾矿库集中整治，坚决遏制重特大事故发生。深化平安朝阳建设，建立完善综合服务管理体系、多元化矛盾纠纷化解体系、激发社会组织活力工作体系、立体化社会治安动态防控体系，不断提升社会治理水平。加强国防教育和民兵预备役、人民防空等工作，搞好第八轮双拥模范城创建，推进军民融合发展。落实民族宗教政策，发展民族团结进步事业。

（九）推进法治、创新、高效和廉洁政府建设

把遵守宪法法律作为政府工作的根本原则，积极构建法治政府治理体系，坚持法无授权不可为、法定职责必须为。全面推进依法民主科学决策和政务公开，严格规范公正文明执法，提高法治思维和依法办事能力。认真落实人大及其常委会的决议、决定，自觉接受人大法律监督、工作监督；深入推进协商民主建设，支持政协全面履行政治协商、民主监督和参政议政职能；认真办理人大代表议案、建议和政协提案。

积极推进工作创新，不断提高工作决策指导能力和攻坚克难、化解矛盾、驾驭复杂局面的能力。

深入抓好软环境建设，坚决纠正懒政、怠政、为官不为以及“只要不出事，宁愿不做事”等消极敷衍行为，提高政府工作效率。

坚持廉洁从政，严守“八项规定”，严纠“四风”，严控“三公”经费支出，严格执行党风廉政建设责任制和廉洁从政有关规定，强化五大系统建设，加强行政效能监察和审计监督，实施公务用车制度改革。

我们要把党的群众路线教育实践活动成果进一步引向深入，努力建设职能有限、行政有为、运转高效的政府，打造一支勤奋、廉洁、公正、有为的公务员队伍。

各位代表，伴随着迈向新征程的坚实足音，我们迎来了充满激情和挑战的2015年。新的一年里，我们将在市委的坚强领导下，与全市各族人民携手并肩，共沐前进风雨，共迎振兴彩虹，用锲而不舍、驰而不息的努力，战胜各种艰难险阻，完成本次会议确定的各项任务，向着新的发展，向着新的胜利，向着建设富裕文明和谐美丽新朝阳的目标奋勇前进！
